# World Journal of Emergency Surgery: "it has been a spectacular beginning"

**DOI:** 10.1186/1749-7922-1-19

**Published:** 2006-06-28

**Authors:** Fausto Catena, Ernest E Moore

**Affiliations:** 1 Consultant Surgeon, General, Emergency and Transplant Surgery DPT, St. Orsola-Malpighi University Hospital, Bologna, Italy; 2 Chief, Trauma Services, Rocky Mountain Regional Trauma Center at Denver Health Medical Center Chief, Department of Surgery, Denver Health Medical, Center Professor of Surgery, University of Colorado Health Sciences Center Vice Chairman, Department of Surgery, University of Colorado Health Sciences Center, Canada

Three months have passed since the WJES was launched. [1] In these few months, we have received a substantial amount of feed-back: 70 papers have been submitted and 10 invited reviews were published. The popularity of the topic has aided the growth and effectiveness of the journal. Now, it is the time to change; the journal is growing and it has "stronger shoulders." [2]

As you know, publishing in WJES has been free of charge, but starting June 24, the journal will introduce the usual Biomed fee. WJES articles will become financially self-sufficient through article-processing charges [APCs]. Authors are asked to pay GBP 750, if their research is accepted for publication. However, authors from resource-poor countries, who are unable to pay the APC for their manuscript, can apply to the Editors-in-Chief to have the APC waived. Other authors, who belong to BioMed Central member organizations, will have the APC covered in full or part by their membership. No charge is made for articles that are rejected after peer review. [2]

Open Access publishing provides instant and universal availability of published work to any potential reader, worldwide, completely free of subscriptions, passwords, and charges. Furthermore, authors retain copyright of their work, facilitating its dissemination. Open Access publishing is made possible by article-processing charges assessed "on the front end" to authors, their institutions, or their funding agencies [3]. With this system, these article-processing charges will pay for three things: first, the electronic submission process, facilitating an efficient and thorough peer review; second, the publication costs involved in providing the article freely and universally in various formats online; and, third, the processes required for the article's inclusion in PubMed and its archiving in PubMed Central, e-Depot, Potsdam and INIST. There is no remuneration of any kind provided to the Editors-in-Chief, to any members of the Editorial Board, or to peer reviewers, as these positions work voluntarily. [4-6]

The benefits from such Open Access will reward readers, with unrestricted access to information; to authors, through the widest possible dissemination of their work; and to science and society in general, by increasing the availability of information and scientific advancement.

We believe World Journal of Emergency Surgery will benefit clinical care and aid scientific research, and we hope you will support this progress by submitting your next research article to this open access journal.
